# Crohn’s Disease Phenotype in a Patient With Severe Congenital Neutropenia Caused by *CSF3R* Variants: Exploring the Pathogenesis and Effects of Thalidomide Treatment

**DOI:** 10.1155/jimr/8163275

**Published:** 2026-07-21

**Authors:** Yanqiu Wang, Lin Wang, Chun Pan, Zhiheng Huang, Ping Li, Xiaoxia Qiu, Cuifang Zheng, Ying Huang

**Affiliations:** ^1^ Department of Gastroenterology, Pediatric Inflammatory Bowel Disease Research Center, National Children’s Medical Center, Children’s Hospital of Fudan University, 399 Wanyuan Road, Shanghai 201102, China, fudan.edu.cn

**Keywords:** *cSF3R*, IBD, phenotype, thalidomide

## Abstract

This study describes a case of severe congenital neutropenia (SCN) harboring biallelic *CSF3R* variants and presenting with a novel inflammatory bowel disease (IBD) phenotype that responded remarkably to thalidomide. We performed functional studies to reveal the pathogenicity of the *CSF3R* and to elucidate the potential mechanisms underlying the therapeutic effects of thalidomide. A comprehensive review of the literature identified 18 reported cases of SCN associated with *CSF3R* variants. We subsequently validated the pathogenicity of the variants in the patient’s peripheral blood by flow cytometry, which demonstrated reduced granulocyte colony‐stimulating factor (G‐CSF) receptor (G‐CSFR) expression and markedly decreased phosphorylation of signal transducer and activator of transcription (STAT)3 (p‐STAT3) following stimulation with recombinant human G‐CSF (rhG‐CSF). Thalidomide augmented rhG‐CSF‐induced STAT3 phosphorylation in HEK293T cells harboring the *CSF3R Q257*  ^∗^ or *R308G/Q257*  ^∗^ variants, and this effect was suppressed by a Janus kinase (JAK)1 inhibitor. Thus, IBD might represent a novel phenotype of SCN caused by *CSF3R* variants, and thalidomide could potentially alleviate the symptoms by modulating the JAK1/STAT3 signaling pathway.

## 1. Introduction

Severe congenital neutropenia (SCN) is a rare myeloid disease characterized by recurrent, potentially fatal bacterial infections and an absolute neutrophil count (ANC) less than 0.5 × 10^9^/L. SCN is caused by variants in several genes, including *ELANE*, *CSF3R*, *GFI1*, *HAX1*, *G6PC3*, *VPS45*, *WAS*, and *JAGN1* [1, 2]. The *CSF3R* gene encodes the granulocyte colony‐stimulating factor (G‐CSF) receptor (G‐CSFR), which belongs to the type I cytokine receptor superfamily, and *CSF3R* variants can cause SCN type 7 (SCN7). The engagement of G‐CSFR triggers a canonical signaling cascade through the Janus kinase (JAK) signal transducer and activator of transcription (STAT) pathway, orchestrating the phosphorylation of STAT1/3/5 to initiate the transcriptional activation of downstream target genes [3]. Previous studies have shown that *CSF3R* variants identified in patients with SCN are predominantly heterozygous and may arise either somatically or de novo during meiosis. These variants typically result in truncated receptor forms and have been associated with leukemogenic progression and a hyperproliferative phenotype in mutant cells [4–6]. Triot et al. [7] first reported inherited biallelic *CSF3R* variants in patients with SCN, and a series of SCN cases caused by germline *CSF3R* variants were subsequently reported. These variants usually affect the extracellular domain of G‐CSFR, unlike somatic *CSF3R* variants, which usually affect the cytoplasmic region of G‐CSFR [8]. Patients with inherited biallelic *CSF3R* variants might exhibit recurrent fever, infections, oral ulcers, diarrhea, and juvenile idiopathic arthritis. Although there were no cases with a clear diagnosis of inflammatory bowel diseases (IBDs) among these patients, some cases showed symptoms of IBD, such as bloody diarrhea, recurrent vomiting, and oral ulcers [9]. In addition, a previous study demonstrated *that Csf3r* ^−^/^−^ mice exhibited increased susceptibility to colitis and colitis‐associated colorectal cancer compared with *Csf3r* ^+^/^+^ mice [10]. These findings suggest that IBD may represent a potential clinical manifestation of inherited *CSF3R* variants. Thalidomide, initially withdrawn in the 1960s but later reintroduced because of its immunomodulatory properties, has demonstrated therapeutic efficacy in several immune‐mediated disorders. Notably, it has shown clinical benefit in pediatric Crohn’s disease (CD), supporting its use as an alternative treatment option in refractory cases and providing a mechanistic rationale for its application in patients with monogenic disorder‐associated CD phenotypes [11]. Here, we report a case of SCN caused by biallelic heterozygous *CSF3R* variants in a patient diagnosed with CD, who showed a significant treatment response to thalidomide. Furthermore, we conducted functional validation on the patient and explored the reasons for the good response to thalidomide treatment.

## 2. Materials and Methods

### 2.1. Patient

The patient with *CSF3R* variants was recruited from the Department of Gastroenterology at the Children’s Hospital of Fudan University (China).

Trio‐based whole‐exome sequencing (WES) was performed on genomic DNA extracted from peripheral whole blood samples of the patient and his parents. Variant interpretation and pathogenicity classification were conducted in accordance with the guidelines of the American College of Medical Genetics and Genomics (ACMG). Comprehensive clinical and genetic data were obtained from the patient’s medical records and standardized clinical registration forms.

Informed consent for participation and blood/intestinal mucosal sample collection were obtained from parents, with approval from the research ethics board at the Children’s Hospital of Fudan University (Approval Number 2023244).

Intestinal mucosal biopsy specimens were obtained during endoscopic evaluation from the patient from inflamed regions as well as from healthy individuals that underwent preventive screening colonoscopy (control subjects).

### 2.2. Protein Structure Modeling

All protein models in this study were visualized using the molecular graphics program PyMol. The homology modeling of G‐CSFR’s structure was built using SWISS‐MODEL (https://swissmodel.expasy.org/interactive) based on the NCBI reference sequence (NP_724781.1). The PyMol mutagenesis program was used to generate the structure of the mutant G‐CSFR. PyMol vacuum electrostatics program was used to compute the electrostatic potential. The Python script “Color_h” was used to color the hydrophobic surfaces according to a normalized hydrophobicity scale (https://pymolwiki.org/index.php/Color_h).

### 2.3. Plasmids and Mutagenesis

The cDNA sequence encoding wild‐type (WT) human CSF3R (NM_156039) was subcloned into the pIRES2‐EGFP‐3Flag vector. Two variants (c.769C > T and c.922C > G) were introgressed using a ClonExpress II One Step Cloning Kit (Vazyme, Nanjing, China). The plasmid sequences were then verified using the Sanger sequencing method. The concentration and quality of the linearized plasmid were confirmed using a NanoDrop Spectrophotometer (Thermo Fisher Scientific, Waltham, MA, USA).

Detailed protocols for plasmid preparation are provided in the Supporting Information, while the validation maps and chromatograms are presented in Figure S1. All mutant cDNA constructs encompassing the full coding regions were verified by the Sanger sequencing prior to their use in subsequent experiments.

### 2.4. Cell Culture and Transfections

HEK293T cells (ATCC CRL‐3216; American Type Culture Collection, Manassas, VA, USA) were maintained in Dulbecco’s Modified Eagle Medium (Thermo Fisher Scientific) supplemented with 10% fetal bovine serum (FBS) (Thermo Fisher Scientific) at 37°C in a humidified incubator containing 5% CO_2_. Cells were routinely passaged at 70%–80% confluence to maintain exponential growth.

Expression of the WT or variant CSF3R channels was achieved by transient plasmid transfection using Lipo8000 Transfection Reagent (Beyotime Biotechnology, Shanghai, China). For the functional experiments, total cDNA (2.5 μg) was transfected. For the co‐transfection experiment, the mass ratio of the two co‐transfected plasmids was 1:1 in 6‐well culture plates. The cells were incubated for 36–48 h before being used in flow cytometry. Cells expressing EGFP were identified by epifluorescence and used for flow cytometry. Detailed step‐by‐step protocols for plasmid transfection are provided in the Supporting Information, while the corresponding sequencing validation reports are presented in Figure S1.

### 2.5. Phosphorylation of STAT3 and Flow Cytometry

**Figure 1:**
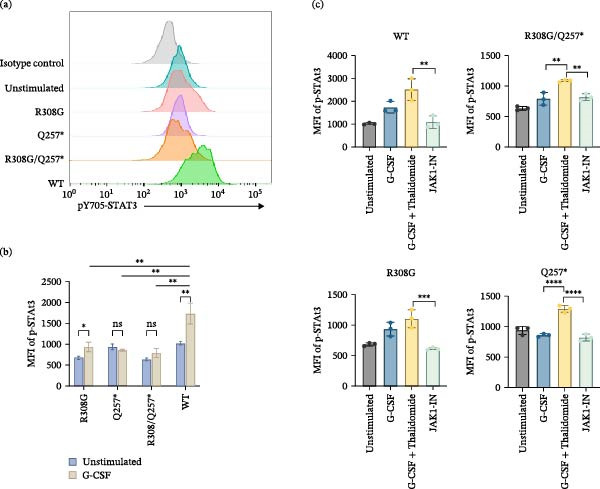
Functional analysis of STAT3 phosphorylation in HEK293T cells transfected with CSF3R constructs. (a) Representative flow cytometry histograms showing p‐STAT3 levels in HEK293T cells expressing wild‐type or mutant CSF3R following cytokine stimulation. (b) Quantification of STAT3 phosphorylation presented as mean fluorescence intensity in HEK293T cells transfected with the indicated constructs; data represent three independent biological replicates (mean ± SEM), and pairwise comparisons between treatment conditions within the same construct were analyzed using an unpaired two‐tailed Student’s *t*‐ test ( ^∗^ *p* < 0.05,  ^∗∗^ *p* < 0.01). (c) Quantification of p‐STAT3 mean fluorescence intensity in HEK293T cells stimulated with G ‐CSF, with or without pretreatment with thalidomide and a JAK1 inhibitor; data represent three independent biological replicates (mean ± SEM) and were analyzed by one‐way ANOVA followed by Tukey’s post hoc test for multiple comparisons ( ^∗∗^ *p* < 0.01,  ^∗∗∗^ *p* < 0.001,  ^∗∗∗∗^ *p* < 0.0001).

Flow cytometry was performed to assess STAT3 phosphorylation and G‐CSFR expression in peripheral blood samples and HEK293T cells. For the analysis of phospho‐STAT3 in human peripheral blood, a direct whole‐blood lysis and fixation protocol was employed, as previously described [12]. Briefly, cells were incubated in a medium containing 5% FBS at 37°C in 5% CO_2_ for 60 min, followed by stimulation with recombinant human G‐CSF (rhG‐CSF) (Cat. No. 300‐23; PeproTech, Rocky Hill, NJ, USA) or recombinant human granulocyte‐macrophage colony‐stimulating factor (rhGM‐CSF) (Cat. No. 300‐03; PeproTech) at final concentrations of 25 and 50 ng/mL, respectively, for 15 min at 37°C. For pharmacological experiments in HEK293T cells (Figure 1c), cells were pretreated with 100 μM thalidomide (Cat. No. HY‐14658; MedChemExpress, Monmouth Junction, NJ, USA) and/or 0.5 μM JAK1 inhibitor (JAK1‐IN‐13; Cat. No. HY‐161015; MedChemExpress) for 60 min prior to cytokine stimulation. The cells were then incubated with fluorochrome‐conjugated antibodies at 4°C for 30 min. After surface staining, cells were fixed using Fix Buffer (Cat. No. 557870; BD Pharmingen, Franklin Lakes, NJ, USA) for 10 min at room temperature. Permeabilization was then performed in Perm Buffer III (Cat. No. 558050; BD Pharmingen, Franklin Lakes, NJ, USA) on ice for 30 min, followed by intracellular staining according to the manufacturer’s instructions. The complete gating strategy is provided in Figure S2. Compensation for spectral overlap was performed during acquisition using single‐stained compensation beads for each fluorochrome. Preliminary assays were conducted using peripheral blood from multiple healthy donors to optimize the protocol. For the final comparative analysis, data from one representative healthy donor processed in parallel with the patient sample are presented.

The following antibodies were used: mouse anti‐human CD114 (CSF3R)‐phycoerythrin (PE) (Clone LMM741; Cat. No. 346106; BioLegend, San Diego, CA, USA), PE‐conjugated isotype control (Clone MOPC‐21; Cat. No. 400112; BioLegend), Alexa Fluor 647 mouse anti‐STAT3 (pY705) (Clone 4/phosphorylation of STAT3 [p‐STAT3]; Cat. No. 557815; BD Pharmingen), and corresponding isotype control (Clone MOPC‐173; Cat. No. 558053; BD Phosflow, BD Biosciences, San Jose, CA, USA).

### 2.6. Histological Analysis

Ileum and colon tissues were embedded in paraffin and cut into sections. The sections were then stained using hematoxylin and eosin (H&E). For immunohistochemical analysis, paraffin‐embedded sections were deparaffinized in xylene and rehydrated through a graded ethanol series. After antigen retrieval, sections were incubated overnight at 4°C with a rabbit anti‐human p‐STAT3 primary antibody (1:500; Cat. No. GB150001; Servicebio Technology Co., Ltd., Wuhan, China). Immunoreactivity was detected using a horseradish peroxidase (HRP)‐conjugated goat anti‐rabbit immunoglobin (Ig)G secondary antibody and visualized with a 3,3^′^‐diaminobenzidine (DAB) chromogen according to the manufacturer’s instructions. The nuclei were counterstained with hematoxylin. Stained sections were examined under a bright‐field light microscope, and histopathological changes were assessed based on established morphological criteria. p‐STAT3 expression was evaluated according to the distribution, staining intensity, and cellular localization of the brown DAB signal.

### 2.7. Literature Review

To identify studies reporting *CSF3R* variants related to SCN, a literature search was carried out in PubMed for relevant articles published until November 23, 2024. All search phrases, including “neutropenia” and “CSF3R” terms, were medical subject heading terms. Each selected article was examined in either the full‐text or abstract form. Duplicate articles, review articles, and abstracts from meetings were not included.

### 2.8. Statistical Analysis

Statistical analyses were performed using GraphPad Prism (version 10.3.0; GraphPad Software, San Diego, CA, USA). Comparisons between the two groups were conducted using an unpaired two‐tailed Student’s *t*‐test. For comparisons involving more than two groups, one‐way analysis of variance (ANOVA) was applied, followed by Tukey’s post hoc test for multiple comparisons. Data are presented as the mean ± standard error of the mean (SEM) unless otherwise specified. A *p*‐value < 0.05 was considered statistically significant. Statistical significance in the figures is denoted as follows:  ^∗^ *p* ≤ 0.05,  ^∗∗^ *p* ≤ 0.01,  ^∗∗∗^ *p* ≤ 0.001, and  ^∗∗∗∗^ *p* < 0.0001. The specific statistical tests used are indicated in the corresponding figure legends.

## 3. Results

### 3.1. Clinical Features of the Patient

A 13‐year‐old male Chinese patient, the second child of non‐consanguineous parents, who presented with recurrent abdominal pain, oral ulcers, and fever, was transferred to our center.

**Figure 2:**
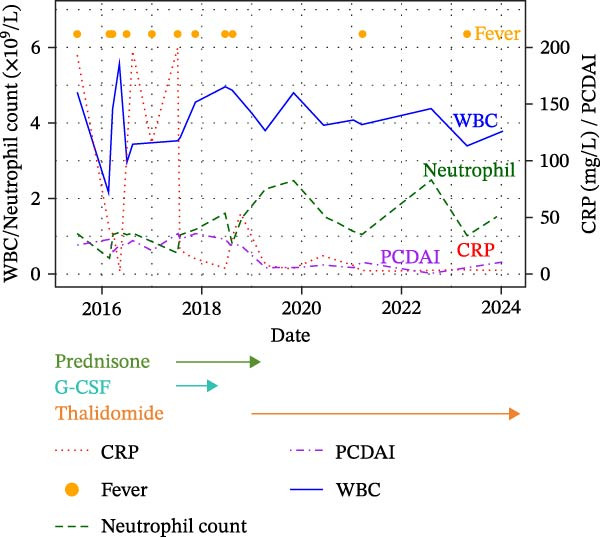
Schematic diagram of the patient’s clinical features and laboratory data before and after treatment.

He was hospitalized for 20 days because of neonatal pneumonia and pleural effusion just 15 days after birth. Subsequently, he suffered from fever of unknown origin approximately every 1–2 months, accompanied by oral ulcers and paroxysmal abdominal pain, with the severity of the abdominal pain gradually increasing. He was diagnosed with SCN at the age of 3, and his ANC to date has ranged between 0.4 and 1.4 × 10^9^/L (Figure 2).

His parents had no history of enteropathy in childhood, and his sibling was healthy. Laboratory examination at the age of 6 showed an increased erythrocyte sedimentation rate and increased levels of C‐reactive protein. Bone marrow examinations showed normal cellularity in terms of the proportion of cells in each granulocyte stage and cell morphology. Several tests to rule out infectious enteropathy were negative, including a T‐SPOT test, detection of Epstein–Barr virus and cytomegalovirus in the blood and intestine, fecal parasite detection, and fecal *Clostridium difficile* testing.

**Figure 3:**
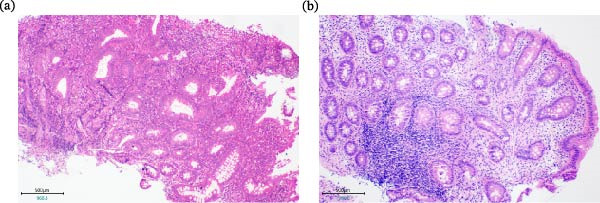
Representative histological images of the patient’s intestinal mucosa. (a) Representative images of H&E staining in the ileocecal region before thalidomide treatment. (b) Representative images of H&E staining in the ileocecal region after thalidomide treatment.

The endoscopic evaluation revealed stenosis of the ileocecal valve and multiple large ulcerations in the ileum and ileocecal valve. Biopsies showed mucosal active inflammation with crypt architectural distortion in the ileocecal region (Figure 3a). Considering the clinical characteristics, laboratory results, endoscopic examination, and histological features, the patient was finally diagnosed as suffering from CD.

**Figure 4:**
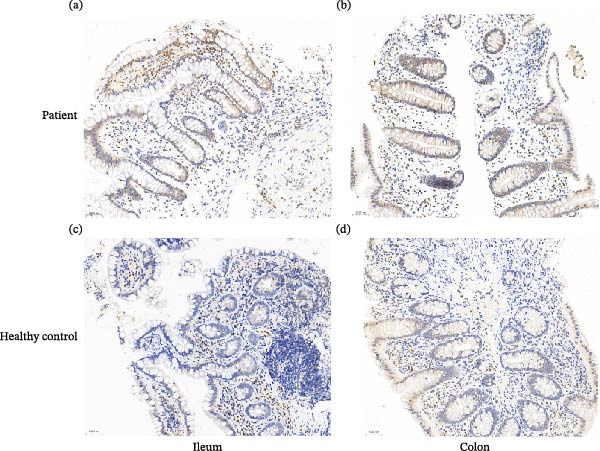
Representative images of p‐STAT3 immunohistochemical staining of the intestinal mucosa from patient’s ileum (a) and colon (b) and the healthy control’s ileum (c) and colon (d).

His symptoms were refractory to mesalamine, corticosteroids, enteral nutrition, and rhG‐CSF. He started taking thalidomide at the age of 7, which significantly alleviated the symptoms of fever and abdominal pain, and the pediatric CD activity index score (PCDAI) dropped from 20 to 40 to below 10. This indicated a transition from active to inactive CD status, with the patient demonstrating a notable clinical response to thalidomide. In addition, his ANC remained between 1 and 2.47 × 10^9^/L, and his white blood cell count was maintained above 3.36 × 10^9^/L (Figure 2). Besides, endoscopic evaluation showed gradual improvement, with the biopsies showing improved active inflammation compared with his previous state (Figure 3b). Immunohistochemistry revealed higher levels of phosphorylated STAT3 in the colon and ileum tissues of the patient compared with those of healthy controls (Figure 4a–d). However, thalidomide was discontinued at the age of 12 because of electromyography, indicating peripheral nerve damage.

### 3.2. Identification of *CSF3R* Pathological Variants in the Patient

**Figure 5:**
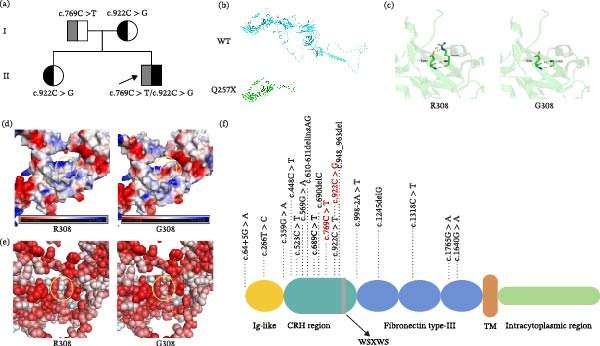
*CSF3R* variants in the patient. (a) The patient’s pedigree. (b) Molecular modeling and predicted structures of the WT protein and the Q257X variant. (c) Changes in the hydrogen bonds and the amino acids of the WT protein and the R308G variant, images display the sticks of relevant residues. (d) The calculated electrostatic potentials of the WT protein and the R308G variant are mapped on the surfaces and colored in a gradient from blue (positive) to red (negative). Yellow circles represent the corresponding amino acids. (e) The predicted hydrophobicity of the WT protein and the R308G variant, shown as gradient colors from white to red to indicate a hydrophobicity scale from low to high. Yellow circles represent the corresponding amino acids. (f) Schematic diagram of the positions of previously identified SCN‐related variants on the CSF3R protein. The locations of the novel variants found in this study are indicated in red.

The proband and his family were subjected to WES, which identified compound heterozygous variants in the *CSF3R* gene (NM_156039) in the patient (Table 1), who inherited a nonsense variant (c.769C > T/p. Q257 ^∗^) from his father and a missense variant (c. 922C > G/p. R308G) from his mother (Figure 5a); in addition, these variants were not found in the Human Gene Mutation Database, gnomAD, or ClinVar.

**Table 1:** The clinical characteristics of the patients in the literature review.

Ethnicity	Sex	Age at onset	Age at diagnosis	ANC (×10^9^L) before treatment	Bone marrow aspiration	rhG‐CSF (µg/kg/d)	rhGM‐CSF (µg/kg/d)	Genetics	Phenotype	Reference
Turkish	M	NA	2.5 y	0.5	Normal	5 (unresponsive)	NA	c.922C >T (hom)	Aphthous stomatitis, bloody diarrhea, recurrent infection	Triot et al. [7] Yılmaz Karapınar et al. [13]
Turkish	F	At birth	At birth	0.42	Normal	5 (unresponsive)	NA	c.922C >T (hom)	Pneumonia, otitis media, recurrent fever	Triot et al. [7] Yılmaz Karapınar et al. [13]
Turkish	F	At birth	At birth	0.41	NA	NA	NA	c.922C >T (hom)	Omphalitis, aspiration pneumonia	Triot et al. [7] Yılmaz Karapınar et al. [13]
Turkish	NA	NA	NA	<0.55	NA	NA	NA	c.1765G >A (hom)	NA	Yılmaz Karapınar et al. [13]
Turkish	NA	NA	NA	<0.55	NA	NA	NA	c.1765G >A (hom)	NA	Yılmaz Karapınar et al. [13]
Turkish	F	9 mo	4 y 3 mo	0.167–1.1	Normal	5 (responsive)	NA	c.266T >C, c.569G >A	NA	Yılmaz Karapınar et al. [13, 14]
Turkish	F	4 mo	3.5 y	0.041–0.508	Normal	15 (unresponsive)	5 qod (responsive)	c.610–611delinsAG (hom)	NA	Yılmaz Karapınar et al. [13, 15]
Parents from Cyprus and England	F	Childhood	44 y	0.5–2.0	Reduced granulocyte progenitors	NA	NA	c.359G >A, c.1640G >A	Recurrent vomiting and fever, mouth ulcers, recurrent infection	Sprenkeler et al. [9]
Turkish	M	At birth	9 y	0.5–1.5	NA	NA	NA	c.1318C >T (hom)	Recurrent fever	Sprenkeler et al. [9]
Turkish	F	Childhood	11 y	0.75–1.75	NA	NA	NA	c.1318C >T (hom)	Recurrent fever	Sprenkeler et al. [9]
NA	F	At birth	At birth	<0.25	Normal	110 (unresponsive)	3 biw (responsive)	c.998‐2A >T, c.1640G >A	Purulent otitis media	Klimiankou et al. [16]
Spanish	F	2 mo	9 mo	0.2–1.0	Normal	40 (unresponsive)	NA	c.948_963del, c.1245delG	Urinary tract infection	Triot et al. [7]
Chinese	F	5 mo	2 y	0.2–0.5	Normal	5 (unresponsive)	3–5 qw (responsive)	c.690delC, c.64 + 5G >A	Recurrent suppurative tonsillitis	Zhou et al. [17]
African Caribbean descent	M	Early childhood	18 y	0.3–1.5	Trilinear hematopoiesis with a subtle left shift in myelopoiesis	NA	NA	c.689C >T (hom)	Anemia, recurrent otitis media, juvenile idiopathic arthritis	Feyen et al. [8]
NA	M	40 d	2 y	0.13–0.7	Hypocellular bone marrow with normal myeloid maturation	5 (responsive)	NA	c.448C >T (hom)	Recurrent fever	Khouj et al. [18]
NA	F	7 y	16 y	0.12–0.60	Mild reduction in myelopoiesis and active other trilineage hematopoiesis	NA	NA	c.523C >T (hom)	Recurrent fever	Khouj et al. [18]
NA	M	3 y	9 y	0.16–0.25	NA	NA	NA	c.523C >T (hom)	Recurrent fever	Khouj et al. [18]
Chinese	M	1 mo	3 y	0.4–2.45	Normal	5 (unresponsive)	NA	c.769C >T, c.922C >G	Crohn’s disease	This study

*Note*: biw, twice a week; qod, every other day; qw, once a week.

Abbreviations: ANC, absolute neutrophil count; F, female; hom, homozygous; M, male; mo, month; NA, not available; rhG ‐CSF, recombinant human granulocyte colony‐stimulating factor; rhGM‐CSF, recombinant human granulocyte‐macrophage colony‐stimulating factor; y, year.

To assess the effects of the two pathological variants on the structure of CSF3R, in silico investigations were conducted. The extracellular domain of CSF3R has the amino acids Gln (Q) and Arg (R) at positions 257 and 308, respectively, which are located in the so called cytokine receptor homology (CRH) region. Among these, the *Q257*  ^∗^ variant introduces a premature stop codon (Figure 5b), whereas the *R308G* mutation results in an arginine‐to‐glycine substitution that alters the hydrogen bonding network with Glu267 (E267) (Figure 5c). In addition, the mutation transformed a neutral Arg to a negative Gly, introducing an opposing charge that would have interfered with interactions with other molecules (Figure 5d) and increased the hydrophobic interactions in this region (Figure 5e).

### 3.3. Literature Review of SCN Cases Caused by *CSF3R* Variants

We conducted a systematic review of the literature published since 2014 to summarize the clinical characteristics and therapeutic outcomes of patients with CSF3R‐associated SCN (Table 1) [7–9, 13–18). To date, 18 kinds of variants in *CSF3R* associated with SCN have been identified globally in 18 patients (Figure 5f). All reported pathological variants of CSF3R are present in the extracellular domain. Among them, c.922C >T is reported the most frequently (6/36, 16.7%), followed by c.1765G >A (4/36, 11.1%), c.523C >T (4/36, 11.1%), and c.1318C >T (4/36, 11.1%). Among the 18 reported patients, 6 were male, 10 were female, and sex was not specified for two individuals. According to the information available, the median age at diagnosis of SCN was 3.25 years, while the median age at onset was 1.5 months. All patients experienced recurring fevers and various types of infections, such as otitis media, tonsillitis, and pneumonia. Among the reported patients, one patient was diagnosed with both juvenile idiopathic arthritis and anemia, while two patients were reported with a combination of gastrointestinal symptoms and oral ulceration, one presenting with hematochezia and the other with recurrent vomiting. The patient reported in this study is the first case to present with a CD phenotype with variations in *CSF3R*. In terms of treatment, nine patients (50%) received varying doses of rhG‐CSF, with only two patients (11.1%) showing positive effects; three patients (16.7%) were administered with different dosages of rhGM‐CSF and experienced varying degrees of improvement. The present study is the first to demonstrate the significant efficacy of thalidomide in treating the disease.

### 3.4. Decreased Levels of G‐CSFR and p‐STAT3 in the Patient With *CSF3R* Pathological Variants

**Figure 6:**
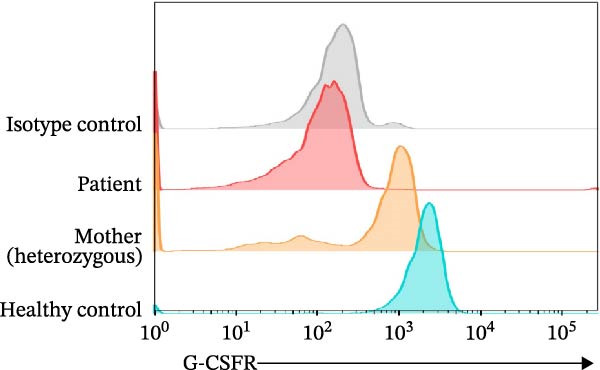
Flow cytometric analysis of G‐CSFR (CD114) expression on peripheral blood neutrophils.

**Figure 7:**
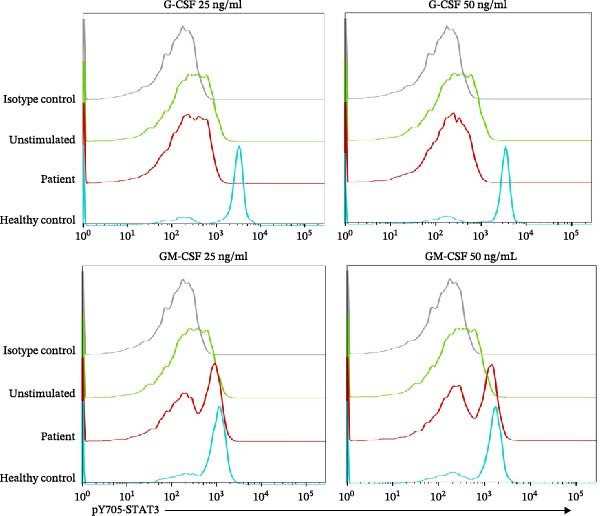
Flow cytometric analysis of STAT3 phosphorylation in peripheral blood neutrophils following stimulation with rhGM‐CSF and rhG‐CSF. Neutrophils from the patient and a representative healthy control were incubated in medium containing 5% FBS at 37°C in 5% CO_2_ for 60 min, followed by stimulation with recombinant human G‐CSF (rhG‐CSF) (25 ng/mL) or recombinant human GM‐CSF (rhGM‐CSF) (50 ng/mL) at 37°C for 15 min prior to intracellular staining for p‐STAT3.

Functional characteristics of the *CSF3R* pathological variants were investigated. Immunostaining for G‐CSFR revealed that the neutrophils had much lower levels, while heterozygous family members had moderate levels (Figure 6). After being incubated with either rhG‐CSF or rhGM‐CSF, the levels of p‐STAT3 were assessed to verify the lack of G‐CSFR signaling. After rhGM‐CSF stimulation, neutrophils from both the control and the patient displayed STAT3 phosphorylation, whereas only the control neutrophils did so following rhG‐CSF stimulation (Figure 7). These findings demonstrated that the patients’ *CSF3R* variants inhibited G‐CSFR signaling.

We next transfected either WT or mutant *CSF3R* constructs into HEK293T cells for functional validation. Following G‐CSF stimulation, the levels of p‐STAT3 in the mutant *CSF3R*‐transfected cells were much lower than those in the WT‐transfected cells, similar to the results in the patient’s cells (Figure 1a,b).

### 3.5. Thalidomide Significantly Enhanced STAT3 Phosphorylation in the Presence of *CSF3R* Pathological Variants

The patient showed significant treatment efficacy with thalidomide. Therefore, we investigated the causes of the patient’s positive reaction to thalidomide medication and performed functional validation. Thalidomide significantly enhanced STAT3 phosphorylation induced by rhG‐CSF stimulation in HEK293T cells upon transfection of the Q257 ^∗^ variant and co‐transfection of the R308G/Q257 ^∗^ variants (Figure 1c). However, no such significant enhancement of STAT3 phosphorylation was observed in HEK293T cells after transfection of the R308G variant alone or in those expressing the wild‐type *CSF3R* (Figure 1c). STAT3 is activated by JAK‐mediated phosphorylation; therefore, we stimulated HEK293T cells with rhG‐CSF after incubating them with thalidomide and a JAK1 inhibitor and observed a substantial reduction in STAT3 phosphorylation (Figure 1c).

## 4. Discussion

G‐CSF, also known as CSF3, is a very important cell growth factor in vivo. It binds to the cell surface‐specific receptor G‐CSFR to activate a complex signal transduction system and exert various biological functions. CSF3R consists of an extracellular region, a cytoplasmic region, and a transmembrane region. The N‐terminal region of the extracellular domain is the IG‐like domain, followed by the CRH region. Between the CRH region and the transmembrane region are three fibronectin type III domains [8, 19, 20]. It is generally believed that the JAK/STAT pathway is the main pathway for G‐CSF signaling, leading to phosphorylation of STAT1, 3, and 5, which can subsequently induce transcription of their target genes. Signaling through CSF3R has been implicated in multiple steps during neutrophil development [3, 9, 21].

Recessively inherited loss‐of‐function *CSF3R* variants can cause SCN; however, they are very rare, and clinical manifestations include recurrent fevers and various types of infections, such as otitis media, tonsillitis, and pneumonia. The general lack of response to G‐CSF in patients with biallelic *CSF3R* variants makes clinical management challenging [13]. Mutations in the *CSF3R* gene that cause SCN also manifest as digestive symptoms. However, to date, the IBD phenotype has not been reported; however, herein, we initially describe a patient who had CD brought on by variants in *CSF3R*. The patient’s neutrophil count dipped as low as 0.4 × 10^9^/L, indicating a neutrophil deficiency. At the same time, he endured recurring episodes of abdominal pain, fever, and oral ulcers. When combined with the endoscopic results, a clear diagnosis of CD was made. To demonstrate the pathogenicity of the *CSF3R* variants in our patient, we first conducted bioinformatic analysis and found that the R308G variant caused changes in amino acid interactions as well as alterations in amino acid hydrophobicity and electric charge. Moreover, the Q257  ^∗^ variant was a protein‐truncating variant. Additionally, using flow cytometry, we discovered that the patients’ neutrophils had lower G‐CSFR expression, which confirmed the pathogenicity of the *CSF3R* variants in our patient.

Normally, CSF3R activation is mediated through G‐CSF. The binding of G‐CSF to CSF3R triggers receptor dimerization, which subsequently induces transphosphorylation of the C‐terminal tyrosine residues. These phosphorylated tyrosine residues then serve as docking sites for multiple downstream signaling mediators, facilitating the formation of specific protein–protein interactions. This molecular cascade ultimately leads to a series of phosphorylation events, including activation of the JAK/ STAT signaling pathway [22, 23]. Previous studies have demonstrated that compared with healthy controls, individuals with SCN carrying *CSF3R* mutations exhibit significantly reduced levels of p‐STAT3 in their peripheral blood neutrophils upon co‐incubation with rhG‐CSF in vitro. However, patients’ neutrophils showed STAT3 phosphorylation upon rhGM‐CSF stimulation [7, 9]. Our experimental data corroborated the aforementioned findings, demonstrating that the identified *CSF3R* variants in our patient lead to both the loss of G‐CSFR membrane expression and the impairment of its biological functions.

Our study represents the first reported case of IBD manifestation in a patient with *CSF3R* variants. IBD represents a chronic inflammatory disorder of the gastrointestinal tract, primarily driven by dysregulation in both innate and adaptive immune mechanisms. The pathogenesis involves compromised innate immune function, which manifests as inadequate regulation of the dysbiotic intestinal microbiota and subsequent hyperactivation of the adaptive immune system. These immunological disturbances collectively initiate a self‐perpetuating inflammatory cascade that ultimately mediates intestinal mucosal injury [24, 25]. Currently, IBD phenotypes have been observed in various inborn error immunity (IEI) diseases, such as variants in *CYBA* and *CYBB* genes that lead to defects in phagocytosis and interleukin 10 (IL10) signaling defects, which lead to defects in immunity [26–28]. In addition, recent advances in neutrophil research have unveiled unprecedented complexity in their involvement in the pathogenesis of IBD, with heterogeneous populations and dual functions, both deleterious and protective, for the host [29, 30]. Collectively, the above findings provide substantial evidence supporting the causal relationship between *CSF3R* variants and the development of IBD phenotypes. In a pivotal study by Carnevale et al. [10], *Csf3r* ^−^/^−^ mice demonstrated significantly enhanced susceptibility to colitis when compared with their *Csf3r* ^+^/^+^ counterparts. Notably, neutrophil adoptive transfer in *Csf3r* ^−^/^−^ mice effectively reversed this phenotypic manifestation. In colitis, the *Csf3r* ^−^/^−^ mice showed increased bacterial invasion and a reduced number of healing ulcers in the colon, indicating that the regenerative capacity of epithelial cells was compromised. Furthermore, the study revealed that neutrophils mediate protection against intestinal inflammation by controlling the intestinal microbiota and driving the activation of an IL22‐dependent tissue repair pathway [10]. This investigation provides compelling evidence supporting the association between *CSF3R* variants and the development of IBD phenotypes at the animal model level. However, in contrast to the findings reported by Carnevale et al. [10], our immunohistochemical analysis of the patient’s colonic and ileal tissues revealed elevated levels of p‐STAT3 compared with those in the healthy controls, whereas *Csf3r* ^−^/^−^ mice exhibited reduced STAT3 phosphorylation in colonic tissues. We hypothesized that this phenomenon could be attributed to either the activation of IL‐22/STAT3‐dependent tissue repair pathways in the intestinal tract following therapeutic intervention [10], leading to upregulated STAT3 expression, or other potential mechanisms that warrant further investigation through the collection and analysis of additional patient cases.

Previous studies have shown that the use of rhG‐CSF significantly improved the prognosis and quality of life of patients with SCN [23, 31]. However, several studies have suggested that biallelic loss‐of‐function variants in *CSF3R* might lead to ineffective treatment with rhG‐CSF [16, 17]. Indeed, our patient showed no response to rhG‐CSF. Interestingly, our patient showed dramatic improvement after thalidomide treatment. After starting thalidomide, the patient experienced significant relief from fever and abdominal pain, and his CD transitioned to a nonactive state. Additionally, his ANC increased between 1 and 2.47 × 10^9^/L. Thalidomide has shown efficacy in the treatment of IBD because of its multifaceted mechanisms of action, which include immunomodulation, anti‐inflammatory effects, and anti‐angiogenic properties. Thalidomide treats IBD by inhibiting pro‐inflammatory cytokines (e. g., tumor necrosis factor alpha [TNF‐α], IL‐6, and IL‐12) and suppressing nuclear factor kappa B (NF‐κB) signaling, thereby reducing intestinal inflammation. It also inhibits angiogenesis, which limits blood supply to inflamed tissues, and modulates T‐cell function by promoting regulatory T‐cells (Tregs) while suppressing pro‐inflammatory subsets (e. g., Th1 and Th17). These actions restore the immune balance and promote mucosal healing, which are key goals in IBD therapy [32–34]. Our study showed that thalidomide enhanced rhG‐CSF stimulated STAT3 phosphorylation in HEK293T cells expressing the Q257 ^∗^ variant or co‐expressing the R308G/Q257 ^∗^ variants. This enhancement could be inhibited using a JAK1 inhibitor, suggesting that thalidomide might exert its therapeutic effects in IBD caused by CSF3R variants by potentiating the JAK1/STAT3 signaling pathway. A study has shown that thalidomide treatment in Bechet’s disease can increase the number of γδ T cells in the later stages of the disease [35]. γδ T cells are key producers of IL‐22, a cytokine that promotes mucosal healing and repairs the intestinal epithelial barrier in humans, thereby alleviating intestinal inflammation [36, 37]. Furthermore, animal experiments have demonstrated that IL‐22 supplementation can alleviate intestinal inflammation in *Csf3r* ^−^/^−^ mice [10]. Therefore, we speculated that thalidomide might exert its therapeutic effects by activating the IL‐22/JAK1/STAT3 tissue repair pathway in our patient. However, the specific therapeutic mechanisms of thalidomide still require further in‐depth research. While thalidomide demonstrated therapeutic efficacy, significant safety concerns persist. Clinical data reveal peripheral neuropathy as the most prevalent adverse event caused by thalidomide (15.6 per 10,000 patient‐months), necessitating treatment discontinuation in 56.8% of affected patients [11, 38]. Unfortunately, our patient also had to discontinue thalidomide because of adverse effects (peripheral neuropathy); thus, the patient’s future treatment plan also requires further contemplation and exploration.

## 5. Conclusions

In conclusion, our research presents a case of CD caused by biallelic *CSF3R* variants, providing a preliminary exploration of the IBD phenotype associated with SCN7 caused by these *CSF3R* variants and the therapeutic effects of thalidomide. However, future research will require the collection of more cases, further investigation into the pathogenesis of IBD caused by *CSF3R* variants, and experimental studies to determine the specific mechanisms of thalidomide in treating patients with IBD with *CSF3R* variants.

## Author Contributions

All authors contributed to the conception and design of the study. Yanqiu Wang collected the clinical data, performed the experiments, and drafted the manuscript. Lin Wang and Ying Huang critically revised the manuscript. Chun Pan contributed to the experimental design and data analysis. Ping Li, Xiaoxia Qiu, and Cuifang Zheng provided clinical data. Ying Huang and Zhiheng Huang were responsible for the clinical diagnosis and related data collection.

## Funding

This study was supported by grants from the National Key Research and Development Program of China (Grant 2023YFC2706501), the Shanghai Medical Innovation Research Special Program (Grant 23Y11905100), and the Shanghai Rising‐Star Program (Grant 22QA1401400).

## Disclosure

All authors reviewed previous versions of the manuscript and approved the final version.

## Ethics Statement

Clinical data were retrospectively collected from medical records and documentation provided by the patient’s parents who gave written informed consent for study participation and biological sample collection. The study was approved by the Ethics Committee of the Children’s Hospital of Fudan University (Approval Number 2023244).

## Consent

Written informed consent for publication was obtained from the patient’s legal guardians.

## Conflicts of Interest

The authors declare no conflicts of interest.

## Supporting Information

Additional supporting information can be found online in the Supporting Information section.

## Supporting information


**Supporting Information** Supporting information methods detailed protocols for the construction and sequence validation of wild‐type and mutant (c.769C>T, c.922C>G) CSF3R expression plasmids, including primer sequences, PCR conditions, recombinant cloning, transformation, plasmid amplification, and extraction steps. Figure S1: Construction and sequence validation of the CSF3R expression plasmids. (a) Schematic representation of the recombinant expression plasmid (pIRES2‐EGFP‐3xFlag‐CSF3R). (b) Sanger sequencing chromatograms confirming the c.769C>T mutation (red box). (c) Sanger sequencing chromatograms confirming the c.922C>G mutation (red box). Figure S2: Gating strategies for flow cytometry analyses. (a) Sequential gating strategy for assessing G‐CSFR (CD114) expression on peripheral blood neutrophils. (b) Gating strategy for analyzing phosphorylated STAT3 (p‐STAT3) in neutrophils after cytokine stimulation. (c) Gating strategy for evaluating p‐STAT3 in transfected HEK293T cells, including selection of singlets and EGFP‐positive cells.

## Data Availability

The datasets analyzed during the current study are available from the corresponding author upon reasonable request.
