# Supplementary material for: Crohn’s Disease Phenotype in a Patient With Severe Congenital Neutropenia Caused by CSF3R Variants: Exploring the Pathogenesis and Effects of Thalidomide Treatment
Source: J Immunol Res. 2026 Jul 21;2026:8163275. doi: 10.1155/jimr/8163275 (PMC13389440; doi:10.1155/jimr/8163275)
Supplement: Supplementary file 1 — Supporting Information Supporting information methods detailed protocols for the construction and sequence validation of wild‐type and mutant (c.769C>T, c.922C>G) CSF3R expression plasmids, including primer sequences, PCR conditions, recombinant cloning, transformation, plasmid amplification, and extraction steps. Figure S1: Construction and sequence validation of the CSF3R expression plasmids. (a) Schematic representation of the recombinant expression plasmid (pIRES2‐EGFP‐3xFlag‐CSF3R). (b) Sanger sequencing chromatograms confirming the c.769C>T mutation (red box). (c) Sanger sequencing chromatograms confirming the c.922C>G mutation (red box). Figure S2: Gating strategies for flow cytometry analyses. (a) Sequential gating strategy for assessing G‐CSFR (CD114) expression on peripheral blood neutrophils. (b) Gating strategy for analyzing phosphorylated STAT3 (p‐STAT3) in neutrophils after cytokine stimulation. (c) Gating strategy for evaluating p‐STAT3 in transfected HEK293T cells, including selection of singlets and EGFP‐positive cells. [file JIMR-2026-8163275-s001.pdf]

Supplementary Methods:

Construction and Validation of Mutant CSF3R Plasmids Mutant Plasmid Construction

The plasmids were constructed in collaboration with Changsha Youbao Biotechnology Co., Ltd.

Primer Design and PCR

Primers were designed as follows:

CSF3R NM_156039 pIRES2-EGFP-3xFlag:

F: CTACCGGACTCAGATCTCGAGGCCACCatggcaaggctgggaaac

R: ATCCTTGTAGTCCATGGATCCgaagctccccagcgcctcc

CSF3R c.769C>T pIRES2-EGFP-3xFlag:

F: ggagccatggTagccaggcctgcacataa

R: ggcctggctAccatggctcccagcacag

CSF3R c.922C>G pIRES2-EGFP-3xFlag:

F: caccctgcagataGgctgcatccgctggcccctgcc

R: tgcagcCtatctgcagggtgtaggcc

The following reaction system was prepared, mixed gently by pipetting, briefly centrifuged, and placed in a PCR instrument. The reaction system and conditions are detailed below:

Reagent	Volume (μl)
ddH_2O	32
10× PCR Buffer	5
dNTP Mix (2.5mM each)	5
$MgSO_4$ (25mM)	3
Forward Primer (10μM)	1.5
Reverse Primer (10μM)	1.5
Template (10ng/μl)	1
KOD-Plus-Neo (1U/μl)	1

Total	50

PCR Reaction Conditions:

Step	Temperature	Time	Cycles
Initial Denaturation	94°C	2 min	1
Denaturation	98°C	10 s	
Annealing	60°C	30 s	30
Extension	68°C	12 min	
Final Extension	68°C	3 min	1
Hold	4°C	∞	1

Recombinant Cloning of PCR Product

The following reaction system was prepared on ice. It was mixed gently by pipetting to avoid bubbles, then briefly centrifuged. The reaction was carried out at 50°C for 15 minutes, then cooled on ice for 5 minutes before immediate transformation.

Recombinant Cloning System:

Component	Volume (μl)
ddH_2O	6
ClonExpress II One Step Cloning Kit	2
Purified PCR Product Fragment	2
Total	10

Transformation Experiment

10 μl of the exchange reaction product was added to 100 μl of competent cells. The tube was gently flicked several times to mix and placed on ice for 30 minutes. A heat shock was applied at 42°C for 90 seconds, followed by incubation in an ice-water bath for 2 minutes. 500 μl of LB medium was added,

and the mixture was placed in a 37°C shaker for 1 hour. An appropriate amount of the bacterial culture was evenly spread on a plate containing the corresponding antibiotic (kanamycin) and incubated upside down in a 30°C incubator for 24 hours.

Plasmid Amplification and Preparation

Colony Picking: 24 hours after transformation, three uniformly sized single colonies were picked and inoculated into 5 ml of LB liquid medium. The final antibiotic concentration in the culture was 100 μg/ml, and it was incubated overnight at 37°C with shaking.

Small-scale Plasmid DNA Preparation: Plasmid DNA was extracted using the TIANGEN Endotoxin-free Plasmid Mini Kit, followed by sequencing verification. The sequencing results were compared and analyzed with the target gene sequence.

Strain Activation

Strain activation was performed in a clean bench, strictly adhering to aseptic techniques.

A sterilized inoculation loop was cooled, then used to dip 10 μl of bacterial culture for streaking on a solid LB plate containing the correct antibiotic.

After inoculation, the plate was placed upside down in a 30°C incubator for 24 hours.

Plasmid Extraction

The culture with the correct sequence was transferred to 10 ml of LB liquid medium containing the corresponding antibiotic and incubated overnight at 37°C. Plasmid extraction was performed using the TIANGEN Endotoxin-free Plasmid Midi Kit. Detailed steps are as follows:

1. Collect the overnight culture in a labeled 5 ml centrifuge tube, centrifuge

at 12,000 rpm for 2 minutes to pellet the bacteria.

2. Discard the supernatant, add 250 μl of Cell Resuspension Solution, and vortex thoroughly to suspend the pellet evenly.
3. Add 250 μl of Cell Lysis Solution, then add 10 μl of Proteinase K, invert 5-6 times to mix gently. Let stand for 1-2 minutes until lysate becomes clear.
4. Add 350 μl of Neutralization Solution, invert to mix thoroughly, allowing proteins to precipitate completely. Incubate on ice for 5 minutes.
5. Centrifuge at 10,000 rpm for 10 minutes, discard the protein precipitate, and collect the supernatant into a new, sterile 1.5 ml EP tube.
6. Centrifuge at 12,000 rpm for 5 minutes. Meanwhile, prepare a labeled binding column. Transfer the supernatant to the column, centrifuge at 12,000 rpm for 1 minute, and discard the flow-through.
7. Add 600 μl of Wash Buffer (pre-prepared), centrifuge at 12,000 rpm for 1 minute, discard the flow-through. Repeat once. Perform a final empty spin at 12,000 rpm for 2 minutes to remove residual buffer.
8. Transfer the column to a new 1.5 ml EP tube in the clean bench, let stand for 10-20 minutes to air dry.
9. Add 95 μl of Nuclease-Free Water to the column, let stand for 2 minutes, centrifuge at 12,000 rpm for 2 minutes. Collect the eluate, label the sample, and proceed with downstream quality control.

The experimental flowchart is shown below:

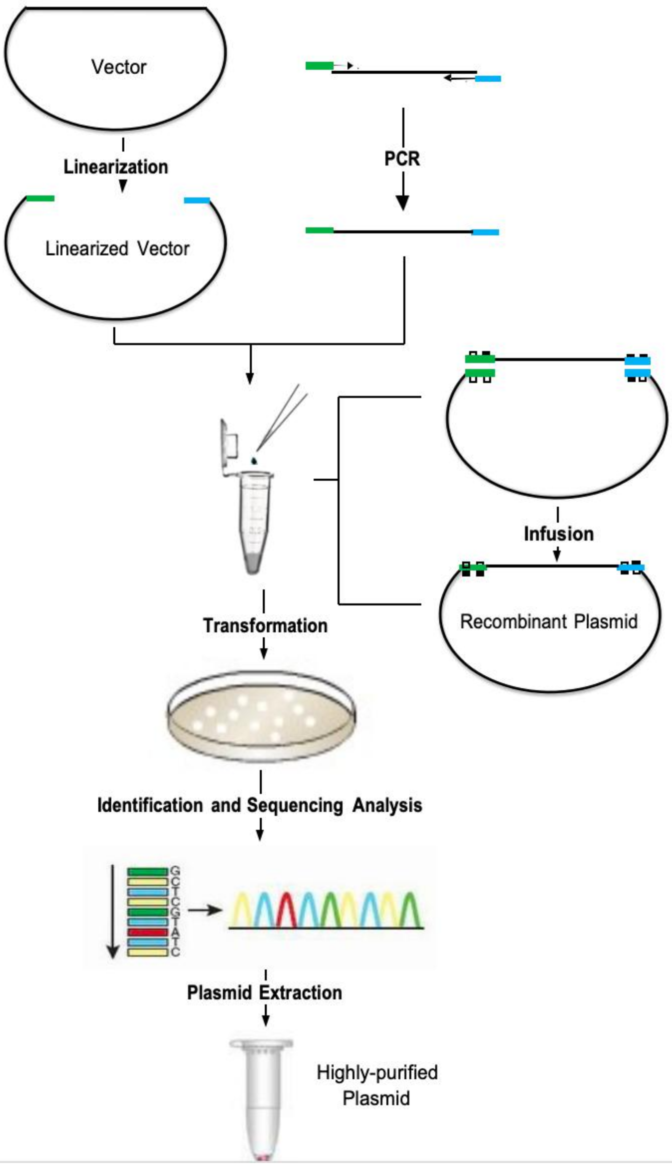

Vector
Linearization
Linearized Vector
PCR
Infusion
Recombinant Plasmid
Transformation
Identification and Sequencing Analysis
G
C
T
C
G
T
A
T
C
Plasmid Extraction
Highly-purified
Plasmid

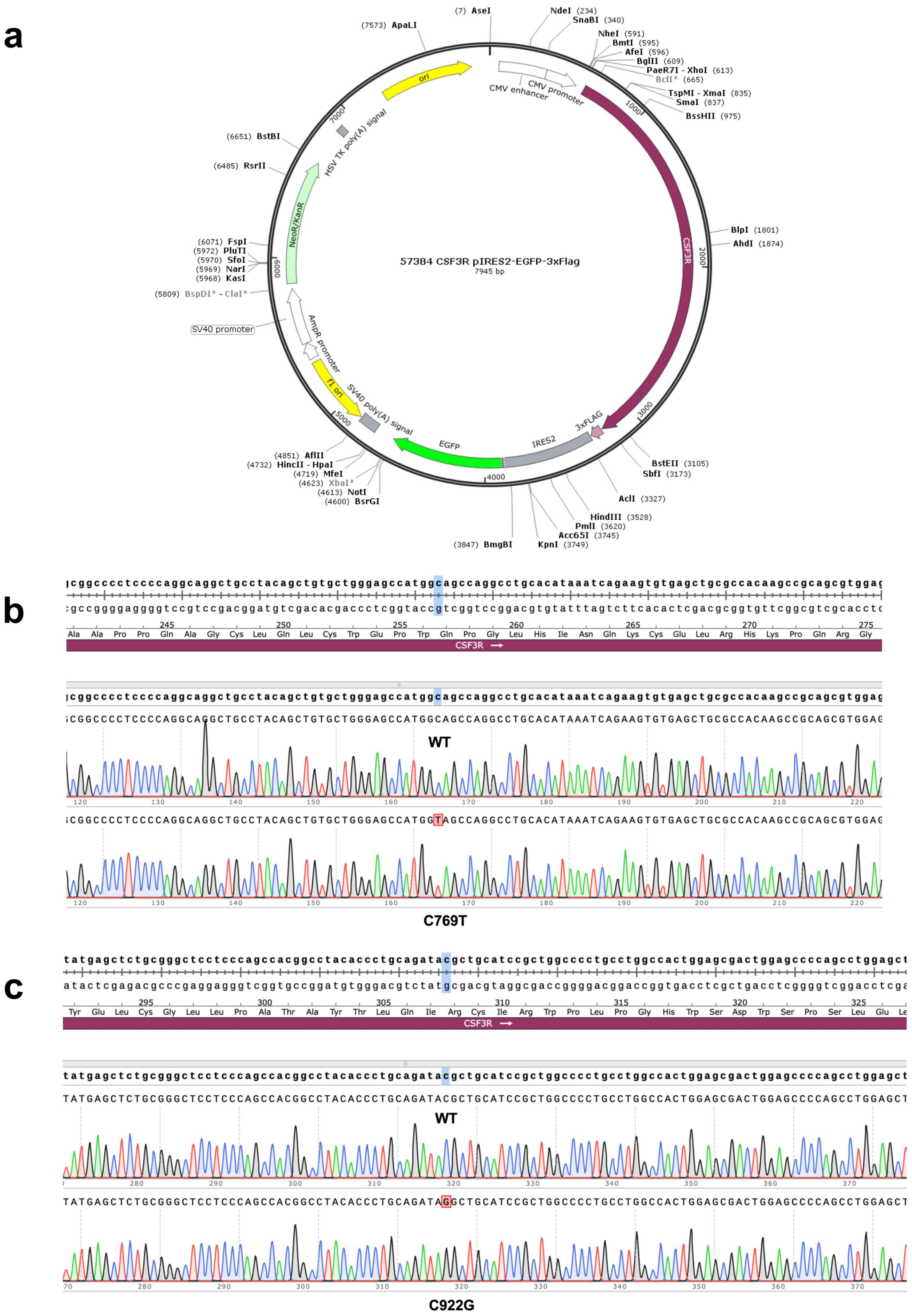

Fig. S1 Construction and sequence validation of the CSF3R expression plasmids.

(a) Schematic representation of the recombinant expression plasmid, harboring the human CSF3R gene cloned into the pIRES2-EGFP-3xFlag vector backbone. (b) Sanger sequencing chromatograms confirming the successful introduction of the c.769C>T mutation. The wild-type (WT) reference sequence is shown in the upper panel, while the mutated sequence is shown in the lower panel. The red box highlights the targeted C to T nucleotide substitution. (c) Sanger sequencing chromatograms confirming the successful introduction of the c.922C>G mutation. The WT reference sequence is shown in the upper panel, while the mutated sequence is shown in the lower panel. The red box highlights the targeted C to G nucleotide substitution.

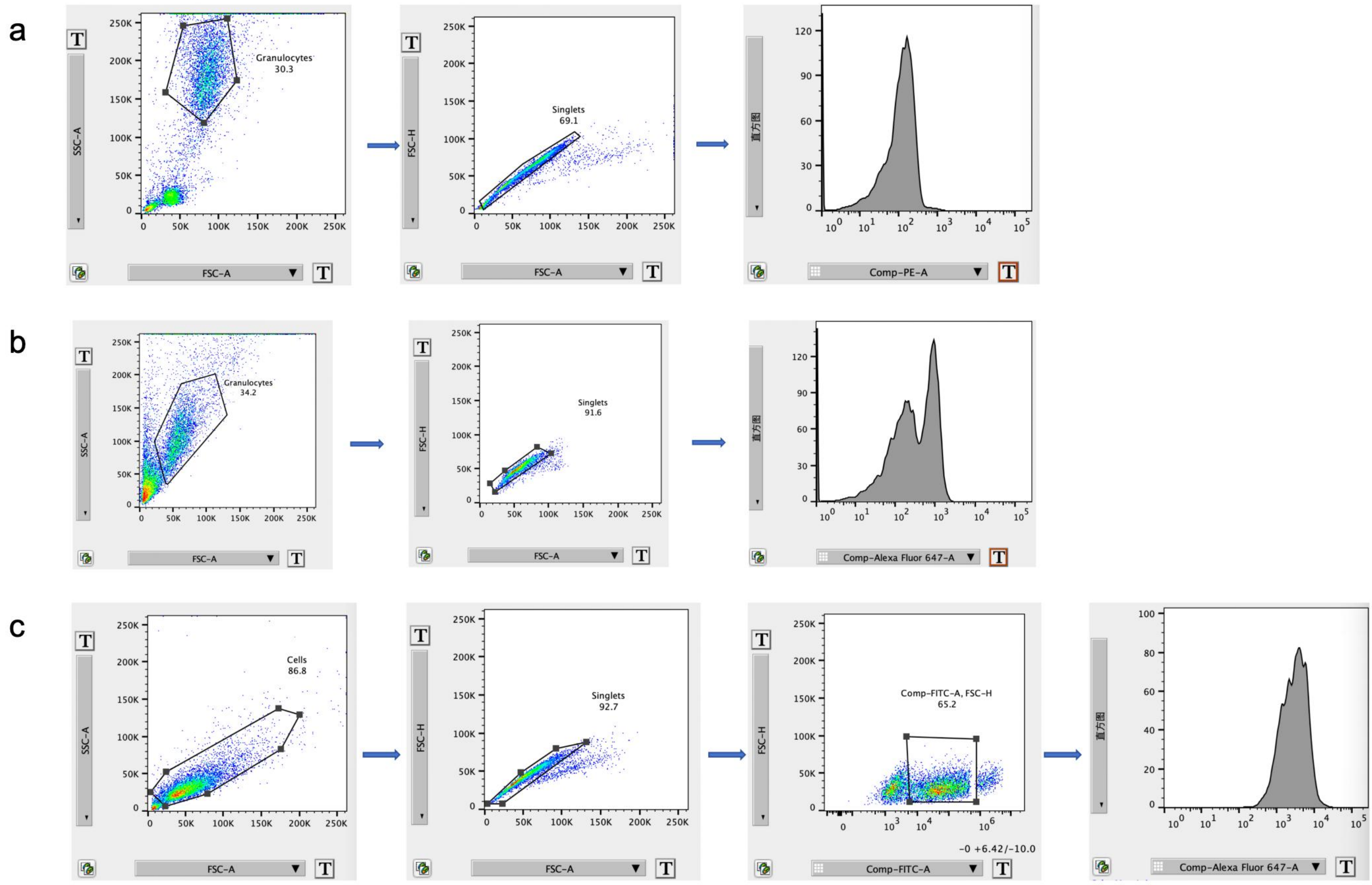

Fig. S2 Gating strategies for flow cytometry analyses. (a) Sequential gating strategy used to assess G-CSFR (CD114) expression on peripheral blood neutrophils. (b) Gating

strategy applied for the analysis of phosphorylated STAT3 (p-STAT3) in neutrophils following cytokine stimulation. (c) Gating strategy used for the functional assessment of p-STAT3 in transfected HEK293T cells, including selection of singlets and EGFP-positive cells prior to intracellular analysis.
